# ACG-EmoCluster: A Novel Framework to Capture Spatial and Temporal Information from Emotional Speech Enhanced by DeepCluster

**DOI:** 10.3390/s23104777

**Published:** 2023-05-16

**Authors:** Huan Zhao, Lixuan Li, Xupeng Zha, Yujiang Wang, Zhaoxin Xie, Zixing Zhang

**Affiliations:** 1 College of Computer Science and Electronic Engineering, Hunan University, Changsha 410082, China; 2 MicroStrategy, Hangzhou 310000, China

**Keywords:** Attn–Convolution neural network, Bidirectional Gated Recurrent Unit (BiGRU), semi-supervised learning (SSL), speech emotion recognition (SER)

## Abstract

Speech emotion recognition (SER) is a task that tailors a matching function between the speech features and the emotion labels. Speech data have higher information saturation than images and stronger temporal coherence than text. This makes entirely and effectively learning speech features challenging when using feature extractors designed for images or texts. In this paper, we propose a novel semi-supervised framework for extracting spatial and temporal features from speech, called the ACG-EmoCluster. This framework is equipped with a feature extractor for simultaneously extracting the spatial and temporal features, as well as a clustering classifier for enhancing the speech representations through unsupervised learning. Specifically, the feature extractor combines an Attn–Convolution neural network and a Bidirectional Gated Recurrent Unit (BiGRU). The Attn–Convolution network enjoys a global spatial receptive field and can be generalized to the convolution block of any neural networks according to the data scale. The BiGRU is conducive to learning temporal information on a small-scale dataset, thereby alleviating data dependence. The experimental results on the MSP-Podcast demonstrate that our ACG-EmoCluster can capture effective speech representation and outperform all baselines in both supervised and semi-supervised SER tasks.

## 1. Introduction

Speech emotion recognition (SER) [1,2], which determines the emotional state of the speakers based on their speech signal, is a fundamental classification task. The classification methods committed to images and text particularly focus on spatially relevant information and temporally contextual information over the data characteristics. Considering the spatiotemporal characteristics of speech data, these methods cannot be used to entirely extract emotion features. This is because speech data have higher information saturation than images and stronger temporal coherence than texts. More specifically, images have two-layer (foreground and background) information, where background information is normally a source of noise to the models, while speech data (also known as spectrograms) have panoramic information. Based on the acoustic characteristics (e.g., intensity and velocity) of speech, there is a strong correlation between any two adjacent elements, while text lacks this property. Semi-supervised methods [3] attempt to learn domain-specific prior knowledge by uncovering the underlying patterns of a vast volume of unlabeled data, thus providing useful general features and structures specific to the data in the supervised model training. In this work, we investigate the challenge of spatial and temporal speech feature extraction over a semi-supervised learning framework, for application to speech emotion classification.

The SER task involves two basic processing steps: speech feature extraction [4], which transforms the original speech signal into lower-dimensional latent features, and speech emotion classification [5], which uses the extracted latent features to satisfy task requirements. In recent years, speech feature extraction technologies have been affected by the boom in computer vision (CV) and natural language processing (NLP) fields, often drawing on some current methods for extracting image spatial features or text temporal features. For example, classic deep learning algorithms such as the Convolution Neural Network (CNN) [6] and the Bidirectional Long- and Short-Term Memory (BiLSTM) [7] are performed to obtain spatial and temporal information, respectively. Although a few very recent studies (e.g., see [8]) have approached a speech-specific spatiotemporal feature extractor with both CNN and autoencoder, these methods either employ an indirect approach that is not designed for speech or lack a scalable framework for data scales. We propose a new framework with an effective speech-specific feature extractor that learns the spatial and temporal features of speech and can be extend to different data scales.

This paper proposes the ACG-EmoCluster, a new semi-supervised framework with an effective speech feature extractor. In particular, our feature extractor integrates an Attn–Convolution neural network and a Bidirectional Gated Recurrent Unit (BiGRU). The Attn–Convolution neural network incorporates the attention mechanism into the convolution processing (Attn–Convolution blocks) for expanding the spatial receptive fields to acquire appearance information of speech frames, which can be extended to arbitrary neural networks with convolution layers and the number of Attn–Convolution blocks that can be scaled for different data scales. The BiGRU obtains the temporal coherence of speech signals and is more conducive to learning on small-scale speech datasets, hence reducing training data requirements. In addition, we are inspired by the semi-supervised learning framework DeepCluster [9], which learns prior knowledge by iteratively clustering speech features using k-means, to enhance the model representation ability. In inference, our model outperforms both supervised and semi-supervised baselines on the MSP-Podcast corpus [10] in terms of the arousal, dominance, and valence for the speech emotion recognition test.

In this paper, ACG-EmoCluster, a novel semi-supervised framework for the SER task, is proposed. It has a feature extractor with an Attn–Convolution network and a BiGRU. The former, which introduces an attention mechanism suitable for speech with panoramic information rather than producing redundant computations, possesses a global spatial receptive field and robust scaling. The latter is more conducive to learning temporal information with a small-scale dataset. By combining the effective feature extractor with a semi-supervised strategy, our model improves the explicit performance of attribute-based SER models in the potential representation.

## 2. Related Work

Speech emotion recognition is an important frontier window in human–computer interaction. It can derive real-time emotional states from interactive data by collecting human semantics, intonation, and other characteristics, thereby improving the fineness and intelligence of the interaction. The current neural networks focus on high-precision performance, paying less attention to the properties of interactive data and the creation of methods that are suitable for these properties. Here, it is very difficult to accurately identify emotions in speech, primarily because of the following reasons: (i) data imbalance results from the fact that most emotional states are neutral and emotional assessments are arbitrary and subjective; (ii) insufficient voice sentiment label data leads to subpar model performance.

In order to extract speech features, the speech’s appearance information and context information must be recorded in both temporal and spatial dimensions. The former is to produce the spatial information of speech frames similar to the image feature extraction techniques in CV and the latter to infer the temporal information of speech sentences such as the autoencoder techniques in NLP. For the task of extracting speech spatial features, Cummins et al. [11] leveraged the AlexNet [12] model to speculate deep spectrum features for the SER task. In [13], an attention pool was designed that can be directly embedded into a deep CNN for speech utterances to enhance the generalization of spatial features. To reduce the convolution computation and memory costs, a novel dilated convolution was structured by [14] that is tuned for standard 1D and 2D filtering and convolution operations. However, these convolution operations must satisfy the requirements of a certain network depth or pooling layer to access global receptive fields. We propose an Attn–Convolution block that introduces global information in convolution without imposing a network depth restriction.

Most studies aim to spot the dependencies between speech states in the temporal dimension. Wang et al. [15], for instance, utilized a BiLSTM to consider the contextual dependencies of speech frames from both forward and backward directions. Fraiwan et al. [16] built a machine learning model with temporal electroencephalogram signals as input to unveil natural interfaces and research into human emotions and responses. Shen et al. [17] deployed a Gated Recurrent Unit (GRU) [18] network that accumulated the speech embeddings into speech representations and reduced the learning parameters. To enhance the model ability and reduce the sample requirements for model learning, we explored the performance of different autoencoders for SER on small-scale datasets.

Semi-supervised learning is made up of two parts: an unsupervised learning process that acquires modality-specific prior knowledge by designing a meaningful objective function and a supervised learning process that initializes the backbone parameters for downstream tasks considering the prior knowledge. In [19], a contrastive semi-supervised learning strategy was proposed, which constructed a contrastive loss for boosting the stability of speech representations. Reconstructing speech data was viewed as an unsupervised goal in [20], as a way to boost the generalizability of the model and advance the expressiveness of speech features. In contrast to unsupervised strategies that employ a sample as the division object and cannot capture class-level information, DeepEmoCluster [9] was made by [21] to refine the unsupervised learning features by specifying a common clustering algorithm for understanding the impact of the labels on a network. Our work also benefits from this semi-supervised learning strategy. More specifically, we collected unlabeled speech data and iteratively grouped the hidden features with a common clustering algorithm, k-means, which is an unsupervised learning process that aims to guide the discriminant ability of the extractor. Then, the subsequent allocation results were utilized as the supervisory object to update the weight of the network.

## 3. Method

**Figure 1:**
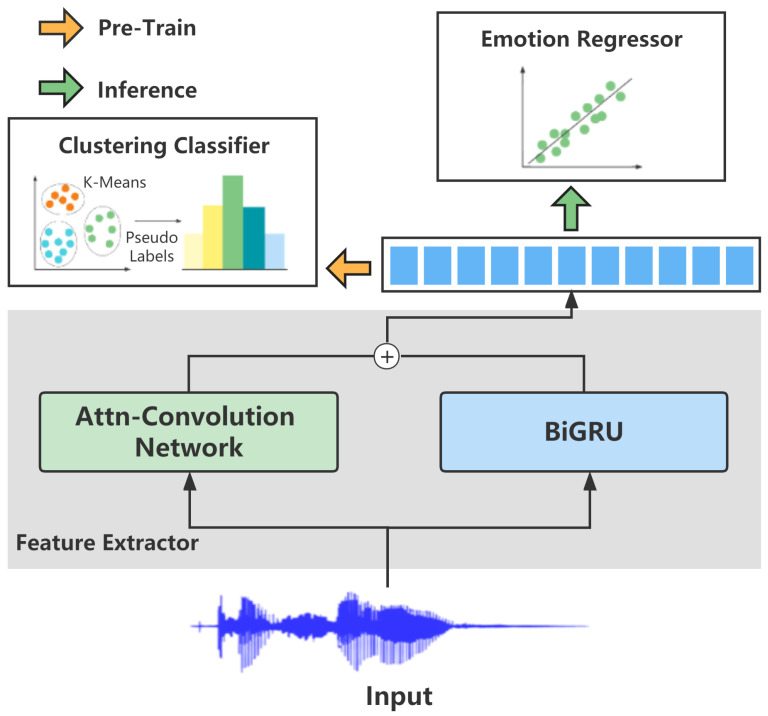
The architecture of our ACG-EmoCluster. During pre-training, unlabeled speech data (e.g., 15 K) are used for learning the prior knowledge of specific speech. During inferring, the backbone initialized by the learned prior knowledge aims to recognize speech emotion.

The goal of our framework, ACG-EmoCluster, was to extract spatial and temporal speech features in parallel for the SER task. Figure 1 shows that it contained two stages: an unsupervised pre-training process to learn the prior knowledge of acoustic data and a supervised SER process to match the speech spatiotemporal features and the emotion labels.

**Speech feature extractor.** The speech feature extractor consists of a spatial feature extraction network and a temporal feature extraction network. As shown in Figure 1, the former was stacked by Attn–Convolution blocks that were modified from the convolution blocks of the VGG-16 [22], and the latter was a BiGRU. The 2D in the VGG convolution be expressed as:

(1) $$z\left(x,y\right)=i\left(x,y\right)\times w\left(x,y\right)=\sum_{a=-c}^{c}\sum_{b=-d}^{d}i\left(a,b\right)\times w\left(x-a,y-b\right),$$

where i(x,y) is the signal input by the model, w(x,y) is the convolution kernel in the convolution, and its size is *c*. Here, the local deep-level speech representation was extracted mainly by modifying the convolution kernel layer by layer. In terms of the size, z(x,y) was the calculation result obtained after the input data were scanned by the convolution kernel, and its size was c×d. Next, this part passed the obtained output value z(x,y) through the convolutional layer to obtain the sequence speech representation:

(2) $$z_{x}^{l}=b_{x}^{l}+\sum_{y}^{}z_{x}^{l-1}\times w_{xy}^{l},$$

where $z_{x}^{l}$ represents the *x*-th speech feature derived from the *l* layer, $z_{x}^{l}$ represents the *x*-th speech feature obtained in the previous layer, that is, the l−1 layer, and $w_{xy}^{l}$ represents the convolution kernel used for calculation between the *x*-th speech feature and the *y*-th speech feature at the *l* layer. The 2D convolutional neural network was mainly composed of the input, a hidden layer, and an output layer and was used to extract local deep spatial features. Specifically, the hidden layer also included a relu layer and a fully connected layer. The convolution kernel is the most important part of the network. It uses a small perceptual window to slide on the spectrogram to gradually condense the emotional characteristics of the speech representation.

**Figure 2:**
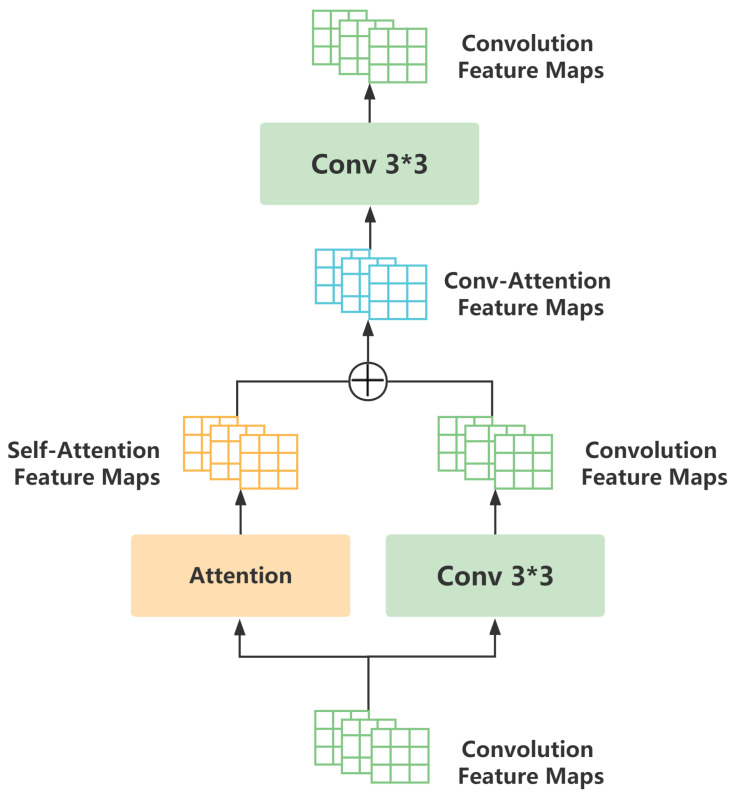
The Attn–Convolution block of the ACG-EmoCluster underwent transformation on the basic convolutional layer. During pre-training, unlabeled speech data (e.g., 15K) were used for learning the prior knowledge of specific speech.

Figure 2 demonstrates that the Attention–Convolution block was made up of two convolution layers and one attention layer. The convolution layer enjoyed a standard convolution with a 3 by 3 kernel, and the attention layer was motivated by the Transformer [23] to incorporate the global information of the feature map into map points. The attention mechanism can be expressed as follows:

(3) $$Attention\left(Q,K,V\right)=softmax\left(\frac{QK^{T}}{\sqrt{d_{k}}}\right)V,$$

where *Q*, *K*, and *V* represent the query, key, and value vectors, respectively, $d_{k}$ represents the dimension of query or key, and $\sqrt{d_{k}}$ represents the scaling factor in attention. The mapping mechanism in the attention function converts a query into a set of key-value pairs, as shown in formula (3). We first determined the dot information for the query vector and all key vectors, divided it by the scaling factor to prevent overcorrelation, and used the Softmax function to determine the weight that corresponded to the value. Finally, we performed point multiplication to determine the output of the attention value. In this way, an effective judgment plan was offered for the model to select more prominent features throughout the training phase when the attention mechanism employed a specific point’s value to determine the relative weights of other feature points in the spectrogram. By adding the global spatial information map to the local spatial features, the model’s power was unavoidably increased. It is worth noting that scaled dot product attention can be computed more quickly and in parallel on the GPU using highly efficient matrix multiplication. As a result, our model selected this attention mechanism to create a quicker and more parameterized scheme with a smaller footprint.

Further, the attention mechanism was tailored for speech frames with panoramic information, which do not incur redundant computations as images do. Differing from general convolution blocks, the receptive field of each map point in the Attn–Convolution block was the entire feature map, instead of 3 × 3. Additionally, we can generalize the Attn–Convolution block to any neural network with the convolution layer and further consider the network depth and data scale to selectively convert the convolution block into the Attn–Convolution block. For temporal feature extraction, we conducted a BiGRU with a few learning parameters to access the contextual information of speech spectrograms and the temporal coherence of latent representations. In practice, we trained the models utilizing both the Attn–Convolution network and the BiGRU network features as speech features.

**Model training.** A classification problem for unsupervised learning and a regression challenge for supervised learning were both included in our model training. The k-means technique was used in the classification task to assign the pseudo labels to unlabeled speech data in order to capture speech-specific prior knowledge. This method of unsupervised learning enabled the model to have general speech characteristics along with data structures. After employing the learned prior information to bootstrap the model’s speech feature extractor, we used a concordance correlation coefficient loss function to fit emotion labels to the SER task. The loss function of the model was represented by the formula (4), where CCC is the consistency correlation coefficient loss, CE is the computed cross-entropy loss for the unsupervised clustering classification, and the parameter λ indicates the significance of the unsupervised task. In this work, we treated them as equals, hence λ = 1.

(4) Loss=1−CCC+λCE.

## 4. Experiments and Results

To evaluate the feasibility and efficacy of the proposed ACG-EmoCluster framework in the speech emotion recognition (SER) task, we compared this framework with the most recent findings. In accordance with [9], we used three metrics in the MSP-Podcast corpus to verify the recognition results: arousal (Aro.), which represents the passive versus active emotional attributes; dominance (Dom.), which represents the weak versus strong emotional attributes; and valence (Val.), which represents the negative versus positive emotional attributes.

### 4.1. Speech Embedding and Preprocessing

This work chose the librosa [24] toolkit to extract the acoustic embedding of the 128D-mel spectrogram from the original speech signal to feed into the ACG-EmoCluster. Our preprocessing included common sampling, pre-emphasis, framing, and windowing, all of which enabled us to disambiguate the effect of lip radiation on the high-frequency section of the voice signal. To be more precise, we first computed the magnitude spectrogram of the waveform signal for a 32 ms frame. The magnitude spectrogram was then used as the input for the 128D Mel-scale filters. The mean and standard deviation of each filter bank’s output were calculated and changed in order to normalize the embeddings. More specifically, the time-domain operations listed below were carried out on the speech signal x[n]:

(5) y[n]=x[n]−αx[n−1],

where α is the pre-emphasis coefficient, which is usually taken as a value close to 1. Additionally, we hoped to retain the connection between frequencies in the time dimension; for this, the windowing procedure is the process of applying the subsequent Hamming window function to each frame:

(6) $$w\left(n\right)=\left\{\begin{array}{cc} 0.54-0.46\cos[2\pi n/(N-1)], & 0\leq n\leq N\\ 0, & others \end{array}\right..$$

Framing, which is comparable to adding a rectangular window to the signal, causes spectrum leakage because the spectrum of the rectangle window produces a huge sidelobe. Therefore, we performed the Fourier transform (FFT) on each frame of the processed speech signal to obtain the power spectrum. The logarithmically transformed mel acoustic spectrogram, in terms of the SER task, exhibited speech characteristics that were more congruent with human emotional expression and more discernible. The spectrogram generated by the Mel filter was transformed by the following equation:

(7) $$m=2595\log_{10}\left(1+\frac{f}{700}\right),$$

(8) $$f=700(10^{\frac{m}{2595}}-1),$$

where *f* represents the frequency of the original speech signal, and *m* represents the Mel frequency value converted by the filter. At the same time, *m* is expressed as the frequency value on the vertical axis in the spectrogram. In the end, we gradually obtained the Mel spectrogram by the above method.

To downgrade the input dimension of the model, we evenly divided the normalized spectrogram into smaller sub-maps using the chunk partitioning method released by Lin and Busso [25]. This method can adaptively adjust the overlap between the spectrogram chunks, that is, segment a speech sequence of any duration into a set of speech chunks with the same number and dimension.

(9) $$\begin{array}{cc} C & =\left\lceil \frac{D_{max}}{t_{c}}\right\rceil , \end{array}$$

(10) $$\begin{array}{cc} \Delta c_{i} & =\frac{D_{i}-t_{c}}{C-1}. \end{array}$$

Equation (1) takes $D_{max}$ as the maximum duration of speech samples on the dataset, $t_{c}$ as the configurable chunk length per sample, and *C*—which works on $D_{max}$ and $t_{c}$ via a ceiling function—as the number of embedding chunks for each sample. The program in Equation (10) calculates the step size $\Delta c_{i}$ between the segmented chunks as a sampling interval of a $D_{i}$-duration-long sample *i*.

Our ACG-EmoCluster accepts identically sized segmented spectrogram sub-maps as input. During model training, we assigned the same emotional labels corresponding to the original sample to all feature sub-maps from the same acoustic embedding. In this study, we averaged the latent vectors of all spectrogram sub-maps after the feature extractor as speech features and their output distributions after the model as the prediction result of speech.

### 4.2. Data and Implementation Details

This work used the MSP-Podcast corpus at version 1.6, a large and common acoustic sentiment dataset, as our emotion corpus. This dataset is a collection of speech fragments from podcast recordings, which was objectively and perceptually annotated by crowdsourcing. It is comprised of 50,362 speech rounds, divided into a testing set with 10,124 samples from 50 contributors; a development set with 5958 samples from 40 contributors; and a training set with 34,280 samples from the remaining contributors. Additionally, we collected 16,044 unlabeled speech samples that were between 1 and 11 seconds long for learning the prior knowledge of the speech data.

For the MSP-Podcast corpus, we used a desired chunk size of 1 second to segment each mel-spectrogram, where the maximum length was 11 seconds, and then collected 11 sub-spectrograms per speech sample. Our model was implemented using Pytorch [26] and was optimized using the Adam [27] optimizer. For the learning rate settings, we initialized the learning rate at 0.0005 for supervised learning and 0.001 for unsupervised learning, and we modified the learning rate following [9]. With a batch size of 32, we put our model into practice while training on an off-the-shelf 2-GPU machine. We used the implementation from [9] with a cluster of 10 for the k-means function. In Table 1, more precise setups are displayed.

As a complement, multi-GPU training benefits from data parallelism and divides each batch of training speeches into multiple GPU batches that are then processed concurrently on each GPU. The final gradient of the whole batch is calculated using the average of the GPU batch gradients. Clearly, training on more than one GPU did not change the outcome in any way. We found that our implementation provided a speed up of 2.36 times on a multi-GPU system in comparison to a single GPU.

### 4.3. Baselines

For comparison, we used several powerful baseline models that are strong in terms of the advantages of semi-supervised learning and feature extractor structure, including:

**CNN-Re** [22]: A regressor and VGG network combination structure that is commonly applied to the extraction of speech features. It incorporates an emotional regression network and a feature encoder, which are only useful for fully supervised learning. We followed the structuring and parameter choices in [9].

**CNN-AE**: Mirsamadi et al. [28] implemented the CNN-AE model by combining convolutional networks with an autoencoder (AE) to alleviate the distribution mismatch problem in speech emotion recognition, providing significant feature representation for the model backbone with the aim of minimizing reconstruction errors. To calculate the negative logarithmic likelihood targets in the data and integrate the feature extraction trunk for improved expression and discrimination, the model simultaneously includes the RBM discriminant approach.

**CNN-VAE**: VAEs, or variational autoencoders, have been quite successful at producing features from real-world data. This prompted Qian et al. [29] to propose the CNN-VAE framework, which makes use of VAEs to improve conventional convolutional networks, makes use of the functional properties of VAEs to derive latent emotional representations in speech signals, and makes use of such representations to categorize emotions.

**DeepEmoCluster**: The DeepEmoCluster [9] model constructs a potential feature space based on emotional content using the deep emotion clustering schema and the VGG framework to extract features from the spectrum. Through joint training based on cross entropy (CE) and consistent correlation coefficient (CCC) double losses, it performs better and achieves higher emotion detection accuracy.

In practice, the CNN-RE trains a standard emotion regressor in the SER task. Its variants, CNN- AE and CNN-VAE, are cluster classifiers; the difference between them is that the CNN-AE reconstructs the low-level feature maps, whereas the CNN-VAE baseline reconstructs the high-level feature maps. Similar to our ACG-EmoCluster, which can be expanded to a supervised framework by randomly initializing model parameters, the DeepEmoCluster is a semi-supervised learning technique. We extracted the experimental results of the comparison methods from the literature [9].

### 4.4. Ablation Studies

We ablated our ACG-EmoCluster using the default setting, as shown in Table 2, Table 3, Table 4, Table 5 and Table 6. Several interesting properties, including the speech feature extractor and unlabeled sample volume, were investigated, with the feature extractor comprising a spatial feature extraction network and a temporal feature extraction network, as well as their combination strategies.

**Spatial network design.** A crucial design of our ACG-EmoCluster was to convert the standard convolution blocks into Attn–Convolution blocks in the VGG-16 network, to enhance speech feature representation. Table 2 details this design. The number of Attn–Convolution blocks of an ACN modified from VGG-16 is important for speech feature extraction. This is different from standard CNNs such as the VGG-16, which can add global information to feature maps in convolution, especially modifying only one convolution layer. ACN-Small (ACN-S), which converts only the first convolution layer of VGG-16, was clearly superior to the conservative (VGG-16) without Attn–Convolution blocks. By modifying the convolution layers even more, the ACN-Large (ACN-L) did not significantly improve the ACN-Base (ACN-B) on Act and Dom. The former converts the first, third, fifth, and seventh convolution layers, while the latter converts all the convolution layers. Overall, the Attn–Convolution blocks we defined were beneficial for enhancing the ability of the speech representations by expanding the perceptual range of the speech feature extraction networks. Owing to this block, a shallow CNN can introduce the global information of the feature map into each convolution.

**Table 2:** Attn–Convolution blocks for spatial information extraction and the ACG-EmoCluster ablation experiments on the MSP-Podcast corpus. We report the SER performance based on a default setting: the speech feature extractor has an Attn–Convolution network with four Attn–Convolution blocks (ACN-B) and a BiGRU network, in which they are combined in parallel, and there are 15 K unlabeled data for pre-training. The default settings and the same parameters as their parts are marked in gray, and the significant results are **bolded**.

Case	Attn–Conv Blocks	Aro. ↑	Dom. ↑	Val. ↑
VGG-16	0	0.6504	0.5400	0.1714
ACN-S	1	0.6556	0.5592	0.1981
ACN-B	4	0.6802	0.5619	0.2217
ACN-L	7	**0.6812**	**0.5632**	**0.2465**

**Sequential network selection.** We compared the different sequential networks, shown in Table 3. The idea in [30] is that the LSTM network and its variants are constrained by the network’s depth and cannot learn high-level features of speech, making it difficult to support the SER task. We borrowed the feature extraction framework of this paper, i.e., a CNN module followed by a LSTM module, to evaluate the various temporal feature extractors. Here, the bidirectional extractors outperformed the unidirectional extractors. This is due to the fact that the bidirectional extractor can aggregate contextual features from speech frames, unlike unidirectional extractors that only perceive features from a single direction. Furthermore, the feature extractor with the GRU was more suitable for learning the dependencies among the convolutional feature maps from the VGG-16. All in all, the feature extractors with the temporal module performed better on the SER task, prominently the BiGRU with bidirectionality and few parameters. The BiGRU as the default sequential network was used to learn temporal features in the following unless otherwise specified.

**Table 3:** Sequential network for temporal information extraction. The default settings are consistent with Table 1. The same parameters and the default settings as their parts are marked in gray, and the noteworthy results are **bolded**.

Case	Aro. ↑	Dom. ↑	Val. ↑
VGG-16	0.6504	0.5400	0.1714
VGG-16+LSTM [s]	0.6579	0.5407	0.1700
VGG-16+GRU [s]	0.6749	0.5696	0.2069
VGG-16+BiLSTM [s]	0.6710	0.5617	0.2074
VGG-16+BiGRU [s]	**0.6752**	**0.5702**	**0.2118**

**Network combination strategy.** We evaluated two strategies for combining the spatial feature extraction network and the temporal feature extraction network. The first strategy, known as parallel [p], entailed the combination of speech features from the original spectrums by the ACN and the BiGRU; the second strategy, known as serial [s], entailed the acquisition of high-level features from the speech spectrums by the ACN first, followed by the introduction of the contextual dependencies by the BiGRU. Table 4 demonstrates that the parallel strategy was superior to the serial strategy for SER. This is because the high-level features extracted from the original speech spectrums through the ACN as the input of the autoregressive network would make it more challenging to detect their dependencies than in the original speech spectrums. In addition, we found that the feature extractor comprising an ACN-B and a BiGRU in parallel achieved the best results. This is due to the model parameter optimization requiring more training samples and iterations when encountering more learnable parameters. With our default settings of 50 training epochs and 15 K of unlabeled data, this instance obtained the optimal performance.

**Table 4:** Combination strategy for generating spatiotemporal information. The default settings are consistent with Table 1. The same parameters and the default settings as their parts are marked in gray, and the noteworthy results are **bolded**.

Extractor	Aro. ↑	Dom. ↑	Val. ↑
ACN-S+BiGRU [s]	0.6824	0.5713	0.2180
ACN-S+BiGRU [p]	0.6835	0.5744	0.2197
ACN-B+BiGRU [s]	0.6882	0.5819	0.2471
ACN-B+BiGRU [p]	**0.6915**	**0.5855**	**0.2542**
ACN-L+BiGRU [s]	0.6842	0.5745	0.2477
ACN-L+BiGRU [p]	0.6831	0.5783	0.2516

**Adequate Training Schedule.** As can be seen from Table 2, when the number of blocks was equal to seven, better results were obtained than when the number of blocks was equal to four. However, contrary to expectations, the combined model with four blocks had the best overall performance, as shown in Table 4. Therefore, we further explored the correlation between the model scale and underfitting and experimented on the model with 100 training epochs. Table 5 shows the experimental results with 100 epochs; here, the model containing seven blocks outperformed the model containing four blocks. The primary cause of this phenomenon is that the 7-block model has larger parameters and requires more fitting epochs. When the training was given enough time, the improved outcomes were obvious. However, it is worth mentioning that the training time of the 7-block model was 2.3 times that of the 4-block model. With insignificant performance differences, we chose four blocks as the basic parameterization of the model, saving computational resources and time costs.

**Table 5:** Performance comparison between the 4-block and 7-block models when training 100 epochs. The default settings are consistent with Table 1. The noteworthy results are **bolded**.

Attn–Conv blocks	Aro. ↑	Dom. ↑	Val. ↑
4	0.6922	0.5861	0.2547
7	**0.6931**	**0.5889**	**0.2628**

**Unlabeled data volume.** The more data, the more effective the model is; there was no exception to this in our ACG-EmoCluster in the SER task. Table 6 depicts the performance of pre-training models with varying scales on the SER task. More unlabeled data resulted in improvements in the arousal and dominance. There was, however, an irregular statistic. This is likely since the acoustic characteristics (such as intensity and velocity) are a perturbation for the division of positive and negative in valence.

**Table 6:** Unlabeled data for learning prior knowledge. The default settings are consistent with Table 1. The same parameters and the default settings as their parts are marked in gray, and the noteworthy results are **bolded**.

Pre-Training Data	Aro. ↑	Dom. ↑	Val. ↑
0K	0.6877	0.5686	0.2373
5K	0.6883	0.5753	0.2409
10K	0.6906	0.5793	0.2303
15K	**0.6915**	**0.5855**	**0.2542**

### 4.5. Results and Analysis

To study the effect of the semi-supervised learning strategy and modules employed in our model, we compared the proposed ACG-EmoCluster with the baselines from different perspectives. Additionally, we compared our model in a poor environment to the DeepEmoCluster in a good environment.

**Supervised Learning.** The results in Table 7 show that our ACG-EmoCluster achieved a dramatic improvement on all three metrics compared with all the supervised learning baselines. For instance, the ACG-EmoCluster increased the arousal score by 0.0375 compared with the DeepEmoCluster and by 0.1291 compared with the CNN-VAE; it increased the dominance score from 0.026 compared with the DeepEmoCluster to 0.0886 compared with the CNN-VAE; finally, it increased the valence score from 0.0547 compared with the CNN-VAE to 0.1019 compared with the CNN-AE. The distinction in the results between the ACG-EmoCluster and DeepEmoCluster (and the others) further proved the superiority of our speech feature extractor.

**Semi-Supervised Learning.** The details of the semi-supervised learning results of SER are shown in Table 7. The results show that by incorporating a successful unsupervised learning strategy before the supervised learning task, both our ACG-EmoCluster and DeepEmoCluster achieved excellent performance: a considerable improvement in all evaluation metrics on the librosa toolkit. By contrast, this illustrates that we developed an excellent speech feature extractor that surpassed all the baselines with semi-supervised learning, particularly DeepEmoCluster (40 K), a semi-supervised method pre-trained on 40 K unlabeled data. Our ACG-EmoCluster (15 K) increased the arousal score from 0.6504 to 0.6915, the dominance score from 0.5400 to 0.5855, and the valence score from 0.1714 to 0.2542 compared to the DeepEmoCluster (15 K) for semi-supervised learning at the same pre-training data size. The obvious margin between the ACG-EmoCluster (15 K) and the DeepEmoCluster (40 K) further proved the superiority of our speech feature extractor and the effectiveness of the semi-supervised learning.

## 5. Conclusions

In this paper, we proposed a novel feature extractor for the SER task inside a framework of semi-supervised learning. While extracting contextual information, this model integrates spatial information to expand the receptive field of the model. This extractor is made up of an Attn–Convolution network for obtaining the spatial information and a low-data-requirement BiGRU for capturing the temporal information. The Attn–Convolution network with an attention mechanism, whose Attn–Convolution block can be extended to any CNN architecture according to the data scale, is better suitable for speech data with panoramic information and does not produce redundant information. It is easily observed that the parallel fusion strategy yielded more effective temporal information from the original acoustic spectrograms than high-level spatial representations. Our proposed feature extractor using fewer unlabeled data achieved a better speech representation than existing semi-supervised frameworks and outperformed the state-of-the-art methods in downstream tasks. Future work will focus on constructing a network that enables the interaction of the spatial and temporal features of speech during training to reduce the computational requirements and enhance the speech representation.

## Figures and Tables

**Table 1:** The experimental configurations of this paper.

Case	Configuration
Development Language	Python 3.11
Deep Learning Framework	Pytorch 1.4.0
GPU	2080Ti
Learning Rate (Adam)	0.0005
Learning Rate (SGD)	0.001
Batch Size	32
CUDA	11.6

**Table 7:** Performance comparison between supervised learning and semi-supervised learning with various SER model architectures. The league table’s noteworthy results are **bolded**.

Method	Pre-Training Data	Aro. ↑	Dom. ↑	Val. ↑
*Supervised Learning*				
CNN-RE [22]	-	0.6177	0.4928	0.1696
CNN-AE [28]	-	0.6338	0.5111	0.1354
CNN-VAE [29]	-	0.5586	0.4800	0.1826
DeepEmoCluster [9]	-	0.6502	0.5426	0.1510
Ours	-	0.6877	0.5686	0.2373
*Semi-Supervised Learning*				
DeepEmoCluster [9]	15K	0.6504	0.5400	0.1714
DeepEmoCluster [9]	40K	0.6611	0.5400	0.1572
Ours	15K	**0.6915**	**0.5855**	**0.2542**

## Data Availability

The data presented in this study are openly available in the MSP-Podcast corpus, at https://ecs.utdallas.edu/research/researchlabs/msp-lab/MSP-Podcast.html, accessed on 29 March 2023.

## Abbreviations

The following abbreviations are used in this manuscript:

SER	Speech Emotion Recognition
BiGRU	Bidirectional Gated Recurrent Unit
SSL	Semi-supervised Learning
CV	Computer Vision
NLP	Natural Language Processing
CNN	Convolution Neural Network
BiLSTM	Bidirectional Long- and Short-Term Memory
CNN-Re	Convolution Neural Network-Regressor
CNN-AE	Convolution Neural Network-Autoencoder
CNN-VAE	Convolution Neural Network-Variational Autoencoder
AE	Autoencoder
VAE	Variational Autoencoder
CCC	Consistency Correlation Coefficient
FFT	Fast Fourier Transform
CPU	Central Processing Unit
GPU	Graphics Processing Unit
CE	Cross Entropy
